# Single-cell atlas of gastric cancer reveals malignant epithelial evolution and regulatory reprogramming of the tumor microenvironment

**DOI:** 10.1371/journal.pone.0347679

**Published:** 2026-04-22

**Authors:** Xiulan Li, Mengqi Guo, Yunhan Wen, Bo Long

**Affiliations:** 1 Department of Gastroenterology, Hunan Provincial People’s Hospital, The First Affiliated Hospital of Hunan Normal University, Changsha, Hunan, China; 2 Research Institute of Lanzhou University in Shenzhen, Lanzhou University, Shenzhen, Guangdong, China; 3 Department Three of General Surgery, The Second Hospital & Clinical Medical School, Lanzhou University, Lanzhou, Gansu, China; Southern Illinois University School of Medicine, UNITED STATES OF AMERICA

## Abstract

The inflammation–intestinal metaplasia (IM)–carcinoma cascade has been proposed as a framework for gastric cancer (GC) development, yet the cell-level heterogeneity and microenvironmental remodeling underlying this progression remain poorly characterized. Here, we constructed a single-cell transcriptomic atlas by integrating scRNA-seq data from chronic gastritis (superficial, CGS), IM, cancer-adjacent, and tumor tissues through a unified analytical pipeline. Seven major cell lineages were resolved. Relative to CGS, IM and GC tissues exhibited a progressive contraction of epithelial compartments accompanied by expansion of immune and stromal populations. Copy number variation (CNV) inference identified two tumor-restricted malignant epithelial subgroups—one biased toward differentiation and the other enriched for inflammatory and epithelial–mesenchymal transition (EMT) signatures—as well as putative proto-malignant intermediates that coexisted with phenotypically normal epithelium. Cell–cell communication analysis indicated broadly augmented crosstalk between epithelial cells and T cells, myeloid cells, and fibroblasts, with prominent involvement of a CD44–extracellular matrix (ECM) axis. Pseudotime trajectory analysis placed malignant epithelium at late positions along gastric and pyloric mucosal cell differentiation backbones, coinciding with increasing CNV burden and enrichment of stem-like transcriptional programs. Gene regulatory network analysis revealed coordinated activity of lineage-specification modules (HNF4/CDX, NR1H4/ESRRA), proliferative regulons (MYC/TFDP1), and inflammatory/EMT-associated programs (FOSL1/REL/NF-κB). In independent cohorts, elevated expression of several malignant-epithelium-associated transcription factors—including HNF4A, KLF3, FOSL1, TCF7L2, BCL3, RELB, ONECUT2, and MAF—correlated with unfavorable overall survival. Collectively, these findings provide single-cell-resolution evidence consistent with the proposed three-stage model of gastric carcinogenesis and highlight candidate transcriptional regulators warranting further investigation as potential early-detection biomarkers.

## 1. Introduction

Gastric cancer (GC) is a high-burden malignancy of the digestive tract worldwide, whose development typically follows the Correa cascade—from chronic superficial gastritis to atrophy, intestinal metaplasia (IM), dysplasia, and ultimately invasive carcinoma [1]. Despite advances in imaging, endoscopy, and molecular testing, overall prognosis has improved only modestly, indicating that key biological events underpinning the transition from early lesions to malignant transformation remain insufficiently understood. Conventional histology and bulk omics technologies provide valuable information at the population-average level but are limited in resolving, at single-cell scale, the fine dynamics of lineage switching, clonal evolution, and remodeling of the tumor microenvironment (TME) during gastric carcinogenesis [2,3]. In particular, precisely when and under what molecular states and ecological contexts malignant epithelial bifurcation emerges from precursor branches remains poorly defined [4,5].

Single-cell RNA sequencing (scRNA-seq) profiles transcriptomes at single-cell resolution, enabling precise delineation of cellular subpopulations, reconstruction of pseudotime/trajectories to trace fate decisions, and inference of intercellular communication through ligand–receptor analysis—a methodological breakthrough for understanding tumor heterogeneity and spatiotemporal evolution [6,7]. In GC, the TME comprises malignant epithelial cells, fibroblasts, and diverse immune cells; among them, cancer-associated fibroblasts (CAFs) and tumor-associated macrophages (TAMs) coordinate via cytokine signaling and extracellular-matrix remodeling to shape immunosuppressive and invasive/migratory phenotypes, and are regarded as critical nodes driving progression and therapeutic resistance [8–10]. However, within a unified framework spanning “superficial inflammation–IM–cancer and adjacent tissues,” integrative studies that systematically elucidate the coupling among epithelial-intrinsic programs, extrinsic microenvironmental signals, and upstream transcriptional regulatory networks remain scarce.

In this study, we integrated scRNA-seq datasets from chronic gastritis (superficial, CGS), IM, GC, and matched adjacent normal tissues. We first used clustering and annotation to precisely locate epithelial populations and combined inferCNV to identify malignant epithelial clones within GC, thereby reducing benign–malignant signal confounding. We then applied cell–cell communication analysis to delineate key interaction axes between tumor-derived epithelial cells and fibroblasts/macrophages; next, trajectory/pseudotime analyses were employed to reconstruct state transitions and branching points from early lesions to malignant transformation; finally, SCENIC was used to resolve transcription-factor networks, defining tumor-specific programs that are activated or repressed, as well as conserved basal epithelial programs. This work aims to address three core questions: (1) whether malignant epithelium arises from specific progenitor branches and the molecular timing and ecological conditions of such bifurcation; (2) whether epithelial–CAF/TAM interaction modules constitute key drivers of proliferation, immune evasion, and matrix remodeling; and (3) which transcription-factor programs govern malignant transformation and could serve as early warning markers and therapeutic targets. Through this integrative strategy, we seek to provide coherent evidence for the cellular ecology and translational biomarkers underlying the initiation and progression of GC.

## 2. Materials and methods

### 2.1 Data selection, quality control, and normalization

To comprehensively characterize the cellular landscape of gastric carcinogenesis, we integrated publicly available scRNA-seq data (GSE150290 [11]) from the Gene Expression Omnibus (GEO) database [12]. The dataset included 51 samples spanning different pathological stages: CGS (n = 3), IM (n = 2), GC (n = 23), and matched adjacent normal tissues (n = 23). As this study involved computational analysis of publicly available, anonymized data with appropriate consent obtained in the original studies, no additional ethics approval was required. We conducted systematic quality control (QC) and normalization, computing transcript counts (nCount_RNA), detected genes (nFeature_RNA), mitochondrial gene fraction, ribosomal protein gene fraction, and hemoglobin gene fraction for each cell [13]. Based on distributional inspection, we retained cells with nFeature_RNA ∈ [200, 5,000] and nCount_RNA ∈ [500, 50,000]; we removed cells with mito_percent ≥ 15% and hb_percent ≥ 3%, and used a lenient lower bound for ribosomal content (percent_ribo > 3%) to exclude putative low-complexity cells [14,15]. At the gene level, we kept features expressed in ≥4 cells to reduce noise. For downstream processing, we applied LogNormalize (scale factor 10,000), selected highly variable genes using the “vst” method (3,000 genes), performed linear scaling (ScaleData), and ran principal component analysis (PCA). Clustering used the first 10 principal components (PCs) with a resolution of 0.1, and UMAP was used for visualization to show the atlas of GC cells [16]. Cell-type annotation combined established markers—epithelial (EPCAM, KRT8), endothelial (VWF, PECAM1), stromal (COL1A1, PTGDS), and immune (PTPRC, CD68, IL1B, CD79A, CD3D, CD3E; mast cell CPA3, TPSAB1) [2,17].

### 2.2 Identification of malignant epithelial cells (inferCNV)

We extracted epithelial cells from GC tumor tissues and implemented a comprehensive quality control workflow prior to Copy number variation (CNV) inference. First, ambient RNA contamination was assessed and removed using decontX (v0.99.3), filtering cells with contamination scores ≥ 0.2. Subsequently, technical doublets were identified and excluded using DoubletFinder (v2.0.3) following parameter optimization via pN-pK sweeping (detailed procedures provided in Supplementary Methods in S1 File). CNV was then inferred at single-cell resolution using inferCNV (v1.24.0) [18]. To construct a high-purity reference, we used T cells from CGS samples with stringent expression criteria (PTPRC > 1 and EPCAM = 0), minimizing contamination by epithelial transcripts. InferCNV was executed with the following key parameters: cutoff = 0.1, cluster_by_groups = TRUE, denoise = TRUE, and HMM = TRUE. Gene coordinates were provided to order features by chromosomes (1–22), enabling chromosome-level aggregation of CNV signals. Following inferCNV normalization and denoising, we performed unsupervised partitioning of the inferCNV expression matrix using k-means clustering. The optimal cluster number (k = 10) was determined by systematically evaluating k = 6–12 through CNV heatmap visualization and CNV score distribution analysis. For each cell, we quantified CNV burden as a CNV score, calculated as the mean squared deviation of the normalized inferCNV signal from baseline (i.e., mean of (expression-1)^2^), which served as an orthogonal, continuous metric of chromosomal imbalance.

Clusters displaying broad, arm-level CNV deviations and elevated CNV scores were annotated as malignant epithelial states (malignant clusters like TumorC1 and TumorC2), whereas clusters whose profiles overlapped the reference distribution were labeled as normal epithelial states (normal clusters like NormalC1 and NormalC2). CNV patterns were visualized using ComplexHeatmap with a three-color gradient scheme (blue for deletions, gray for normal, red for amplifications at values 0.4, 1.0, and 1.6, respectively). Final inferCNV-based labels and per-cell CNV scores were merged back into the unified Seurat object for downstream visualization and integration with trajectory, cell–cell communication, and transcription-factor network analyses. Detailed analytical procedures, parameter settings, and complete R code are provided in Supplementary Methods in S1 File and Supplementary Code in S2 File.

### 2.3 Cell–cell communication analysis (CellChat)

Cell–cell communication was inferred using the CellChat R package following the authors’ recommended default workflow [19]. From tumor tissues we subset four major compartments—epithelial cells, fibroblasts, myeloid cells, and T cells—and constructed a group-level signaling network to quantify interaction strength and interaction counts among these cell classes. Analyses used the human ligand–receptor database bundled with CellChat; overexpressed genes and interactions were identified, communication probabilities were computed and filtered using default thresholds, and networks were aggregated at both pathway and pair levels. Results were visualized with circle/circle-chord graphs (global interaction strength/number) and bubble plots (ligand–receptor pairs and pathway-level differences) [16]. To assess malignant epithelium–specific communication, epithelial cells were further stratified into inferCNV-defined malignant versus non-malignant epithelial subpopulations. We then recomputed communication probabilities within tumor samples and compared outgoing (sender) and incoming (receiver) signaling strengths between malignant and non-malignant epithelium against fibroblasts, myeloid cells, and T cells. Pathway information flow and centrality metrics were summarized to highlight signaling programs most altered in malignant epithelium. Complete R code are provided in Supplementary Code in S2 File.

### 2.4 Developmental trajectory of malignant epithelium (Monocle2)

Pseudotime reconstruction was performed with Monocle2 using the package-recommended workflow [20,21]. Epithelial cells from tumor tissues were used to build a CellDataSet; size factors and dispersions were estimated, and ordering genes were selected by Monocle2’s dispersion-based criterion (from dispersionTable), requiring mean_expression ≥ 0.1 and dispersion_empirical ≥ dispersion_fit. The ordering filter was set accordingly, followed by dimensionality reduction with DDRTree and cell ordering with orderCells to obtain pseudotime and state assignments. To orient the trajectory from putative non-malignant progenitors toward malignant endpoints, the root was initialized in inferCNV-negative epithelial subsets (adjacent/precancer contexts) when available. Monocle2 state labels were integrated back into the Seurat object and used to identify state-specific marker genes with FindAllMarkers (Wilcoxon, FDR-adjusted P values, P_adjust_ < 0.05). In parallel, dynamic genes along pseudotime were queried with Monocle2’s differential testing across states/pseudotime. Functional interpretation of state markers and pseudotime-associated genes was performed via gene ontology (GO) and Kyoto Encyclopedia of Genes and Genomes (KEGG) enrichment (Benjamini–Hochberg FDR correction, P_adjust_ < 0.05) [22,23]. Per-sample analyses and metadata harmonization were used to assess robustness across individuals.

### 2.5 Transcription factor regulatory network analysis (SCENIC)

Using the epithelial-cell expression matrix and corresponding metadata, we applied the standard SCENIC workflow to construct and evaluate transcription factor (TF) regulatory networks [24,25]. Briefly, lowly expressed genes were removed with geneFiltering, gene–gene correlations were computed, and a co-expression network was inferred with GENIE3. We then performed cis-regulatory motif enrichment with RcisTarget using the human motifAnnotations_hgnc_v9 and a local cisTarget database to generate TF-centered regulon sets. Per-cell regulon activity was quantified with AUCell to obtain AUC scores, which were subsequently binarized (ON/OFF) using data-driven thresholds to yield robust activity states. For visualization, both the AUC and binarized activities were integrated into the Seurat object’s metadata to produce UMAP overlays, violin/ridge plots, and heatmaps. In parallel, based on cell-type or subcluster annotations, we calculated the Regulon Specificity Score (RSS) to quantify the specificity of each regulon across epithelial subpopulations. Complete R code are provided in Supplementary Code in S2 File.

### 2.6 External validation of TFs

#### 2.6.1 RNA-seq data for GC.

To validate the clinical relevance of identified TFs, we performed external validation analyses using publicly available datasets and online bioinformatics platforms. Differential expression of candidate TFs between GC tissues and normal gastric tissues was evaluated using the Gene Expression Profiling Interactive Analysis (GEPIA) database (http://gepia.cancer-pku.cn/) [26]. GEPIA integrates RNA-sequencing data from The Cancer Genome Atlas (TCGA) and the Genotype-Tissue Expression (GTEx) project, providing comprehensive gene expression profiles across multiple cancer types [27,28]. For each target TF, we retrieved expression data from gastric adenocarcinoma (STAD) tumor samples (n = 408) and matched normal gastric tissues (n = 211). Expression levels were compared using |log2FC| > 1 and q-value < 0.01 as significance thresholds. Results were visualized as bar plots, with statistical significance indicated by p-values derived from one-way ANOVA. This analysis allowed us to determine whether TFs identified in our scRNA-seq datasets exhibited concordant expression patterns at the bulk tissue level.

To comprehensively assess the prognostic value of candidate TFs, we performed survival analyses using two complementary platforms: Kaplan-Meier (KM) Plotter and GEPIA3 [29]. KM Plotter integrates gene expression data with clinical outcomes from 875 GC patients across multiple independent cohorts, while GEPIA3 provides survival data based on the TCGA-STAD cohort. For both platforms, patients were stratified into high- and low-expression groups based on median expression cutoffs, and overall survival (OS) differences were evaluated using the log-rank test. Hazard ratios (HR) with 95% confidence intervals (95% CI) and log-rank p-values were recorded for each TF. Results were visualized using forest plots.

#### 2.6.2 scRNA-seq for GC.

We further performed an independent validation using the GC scRNA-seq dataset GSE163558 [30]. This dataset comprises samples from one normal tissue, three primary tumors, two lymph node metastases (LNM), two liver metastases, one ovarian metastasis, and one peritoneal metastasis, totaling 53,338 cells, including 9,675 epithelial cells. First, cell identities were annotated across all cells, and epithelial cells were subsequently extracted for downstream analyses. Epithelial cells derived from the normal tissue were used as the reference to conduct inferCNV analysis for evaluating copy-number alteration profiles. Based on the inferCNV-inferred results, DotPlot was applied to validate the expression patterns of transcription factors identified in the preceding analyses across different groups (primary tumors and distinct metastatic sites). The quality-control workflow and key procedures and parameter settings for inferCNV were kept identical to those used in the prior analyses to ensure comparability and robustness.

## 3. Results

### 3.1 Single-cell atlas of GC and features of malignant epithelium

After stringent QC and data integration, a total of 215,038 cells were retained for clustering. Cells resolved into seven major lineages—epithelial, fibroblast, endothelial, myeloid, T cells, B cells, and plasma cells—with consistent and reproducible cluster architecture across cancer-adjacent tissue, tumor tissue, CGS, and IM cohorts (Figure S1A in S3 File). In terms of composition, relative to CGS, B/T cells, myeloid cells, and fibroblasts increased, whereas epithelial cells markedly decreased in IM and GC (both tumor and adjacent) samples, with the most pronounced shift observed in tumors (Figure S1B in S3 File). This “epithelial contraction alongside immune/stromal expansion” indicates that, along the progression from superficial inflammation and IM to carcinoma, the immune-inflammation axis and stromal-remodeling axis increasingly dominate the tissue ecosystem. Molecularly, heatmaps showed lineage-matched, mutually distinct marker signatures for each compartment (Figure S1C in S3 File), and bubble plots of representative markers further validated annotation accuracy and biological plausibility (Figure S1D in S3 File).

**Fig 1:**
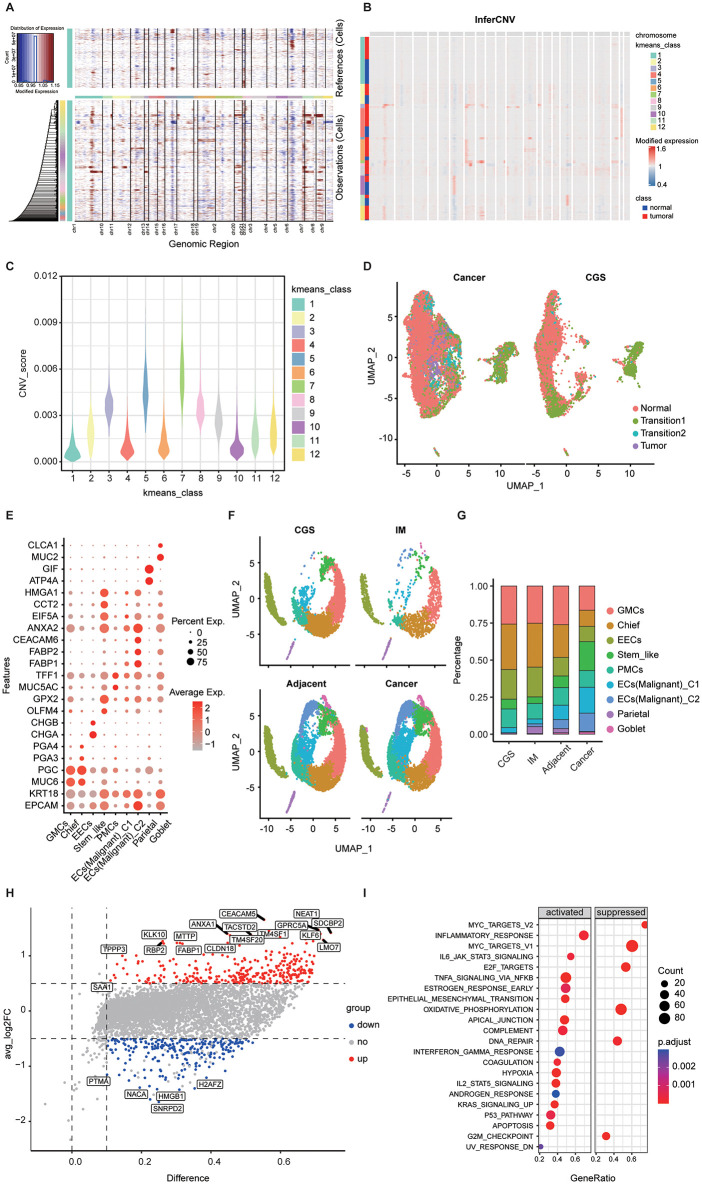
Inference and characterization of malignant epithelium in gastric tumors. **(A)** inferCNV profiles of epithelial cells with T cells from CGS as the reference (PTPRC > 1 and EPCAM = 0). X-axis indicates chromosomes; red within the heatmap denotes higher CNV. **(B)** k-means clustering of inferCNV signals. **(C)** CNV scores per cluster. Clusters with the lowest scores and enrichment of reference cells are annotated as normal; clusters with the highest scores comprised exclusively of tumor epithelium are annotated as malignant; intermediate clusters containing both reference and tumor cells are annotated as transitional (proto-malignant) given the CGS-like inflammatory background. **(D)** UMAP showing the distribution of CNV-based epithelial identities. **(E)** Marker-gene features of epithelial subclusters (red indicates higher expression). **(F)** Distribution of epithelial subclusters across the four tissue contexts. **(G)** Proportions of epithelial subclusters by tissue type. **(H)** Volcano plot of differentially expressed genes between the two malignant epithelial groups (C1 vs. C2) in tumors. **(I)** GSEA of differentially expressed genes between malignant epithelial groups. CGS, chronic gastritis (superficial); IM, intestinal metaplasia; EECs, enteroendocrine cells; PMCs, pit mucous cells; GMCs, gastric mucous cells.

Epithelial cells were extracted from gastric tumor tissues. After removing ambient RNA contamination and doublets, 9,218 tumor epithelial cells and 602 T cells (as reference) were subjected to inferCNV analysis. The results revealed high-amplitude CNV signals in epithelial cells from gastric tumors (Fig 1A). K-means clustering of the CNV matrix (k = 12) partitioned cells into 12 CNV-defined clusters (Fig 1B), some tumor-exclusive and others overlapping with the reference. CNV scores (Fig 1C) delineated three strata: Clusters 1, 4, 6, and 10 with the lowest CNV burden and overlap with CGS-T references, annotated as normal epithelial states; Clusters 3, 5, 7, and 8 with the highest CNV burden, almost entirely composed of tumor epithelium, annotated as malignant epithelial states; Clusters 2, 9, 11, and 12 with intermediate CNV, partly overlapping CGS-T, annotated as evolving (proto-malignant) states, consistent with the inflammation-dominated milieu of GC and its partial resemblance to CGS. Projection onto the epithelial UMAP (Fig 1D) confirmed that malignant epithelium was confined to tumors, whereas normal and evolving states occurred in both tumors and CGS. Reclustering epithelial cells across the four tissue contexts yielded nine epithelial subclusters (Fig 1E and 1F). Malignant epithelium, as defined by inferCNV, formed two tumor-enriched groups that were spatially distinct from epithelial clusters in CGS/IM. In proportion, tumors exhibited not only marked enrichment of malignant epithelium but also a notable expansion of stem-like epithelium (Fig 1G). Differential expression between the two malignant groups (C1 vs. C2) showed that C2 preferentially upregulated inflammatory responses, tumor-antigen presentation, and transcriptional programs associated with epithelial–mesenchymal transition (EMT), whereas C1 retained more epithelial differentiation features (Fig 1H). Gene Set Enrichment Analysis (GSEA) corroborated activation of inflammatory pathways and EMT in C2 (Fig 1I). Together, these data portray malignant epithelium as a continuum spanning epithelial-like to inflammation/EMT-enhanced endpoints.

### 3.2 Cell–cell interaction landscape in tumors

**Fig 2:**
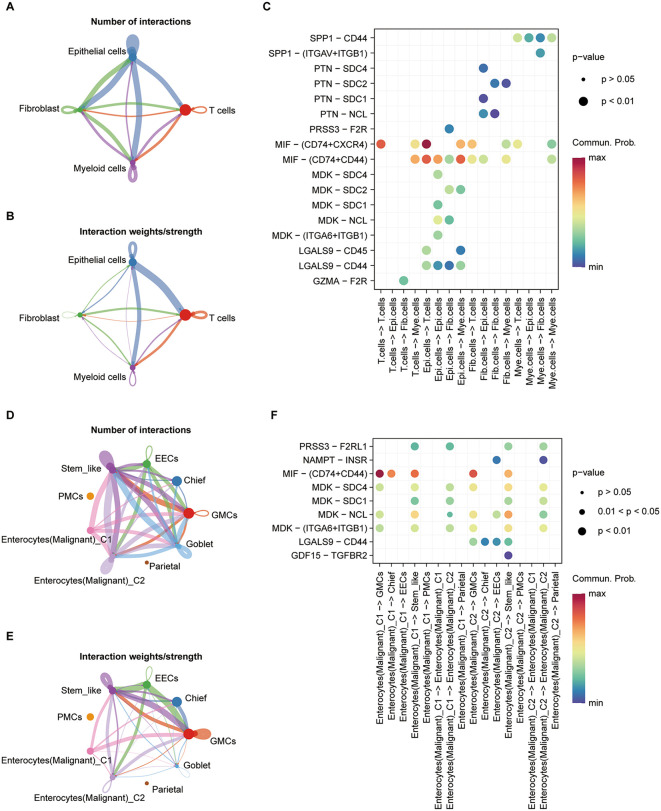
Cell–cell communication among epithelial, fibroblast, T-cell, and myeloid compartments in tumors, and within epithelial subtypes. **(A–C)** For all tumor epithelial cells: **(A)** number of interactions, **(B)** interaction strength, and **(C)** ligand–receptor pair profiles with fibroblasts, T cells, and myeloid cells. **(D–F)** Within epithelial subtypes: **(D)** number of interactions, **(E)** interaction strength, and **(F)** ligand–receptor pair profiles. CGS, chronic gastritis (superficial); IM, intestinal metaplasia; EECs, enteroendocrine cells; PMCs, pit mucous cells; GMCs, gastric mucous cells; Epi, epithelial cells; Mye, myeloid cells; Fib, fibroblasts.

CellChat analysis among tumor-resident epithelium, fibroblasts, T cells, and myeloid cells (Fig 2A–2C) showed a global increase in interaction numbers between epithelium and all partners (Fig 2A). By interaction strength, epithelium–T cell connections were the strongest (Fig 2B), underscoring the centrality of the immune axis. At the ligand–receptor level Fig 2C), the CD44 signaling axis dominated epithelial communications with T cells, myeloid cells, and fibroblasts (details in Fig 2C), consistent with enhanced adhesion/migration and extracellular matrix (ECM)–derived cues. Within epithelial subtypes, the stem-like epithelial subset displayed both higher interaction counts and strengths (Fig 2D and 2E), with active bidirectional information flow to malignant epithelium, intestinal endocrine cells (EECs), and gastric mucous cells (GMCs). Ligand–receptor patterns pointed to growth-factor, ECM-remodeling, and chemokine modules as comparatively reinforced (Fig 2F). These features dovetail with the observed ecosystem reorganization (epithelial contraction with immune/stromal expansion) and provide an ecological rationale for the trajectory and regulatory rewiring described below.

### 3.3 Evolutionary features of tumor epithelium

**Fig 3:**
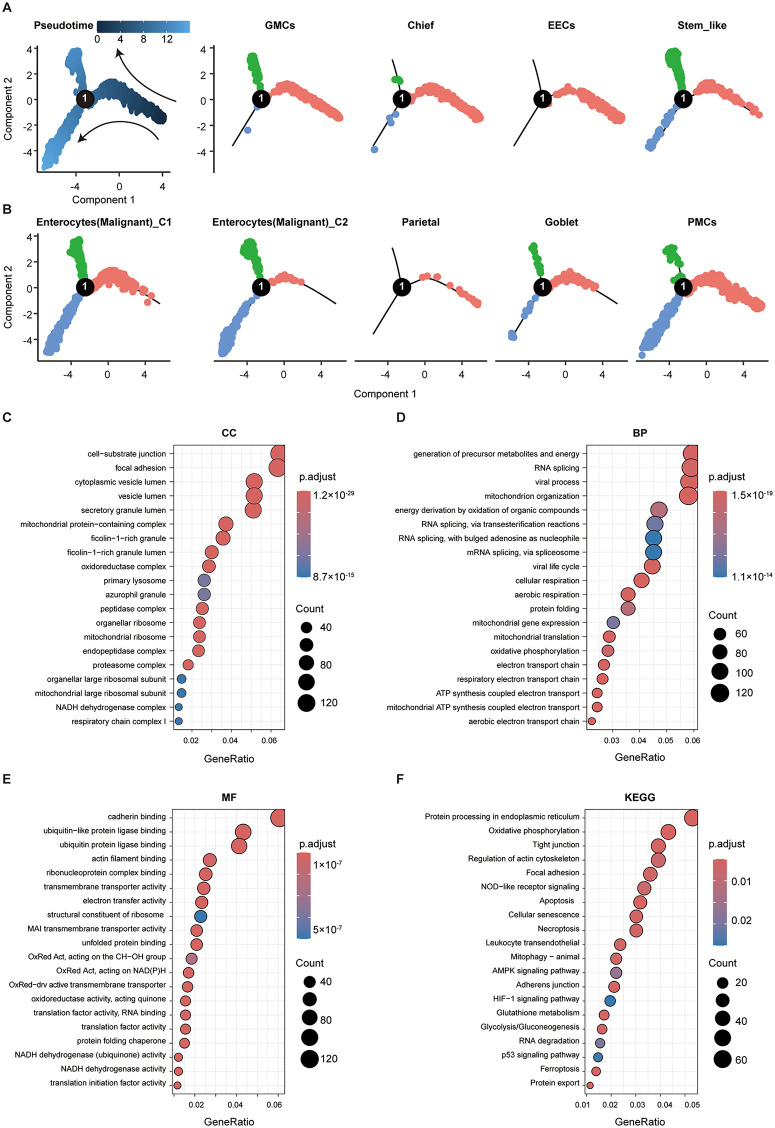
Developmental trajectory features of tumor epithelium. **(A–B)** Monocle2 pseudotime trajectory of tumor epithelial cells and the distribution of epithelial subclusters along the trajectory (dark blue = early stage; light blue = later differentiation). **(C–F)** Functional enrichment of state-specific genes from malignant epithelium: **(C)** cellular components, **(D)** biological processes, **(E)** molecular functions, and **(F)** KEGG pathways. CGS, chronic gastritis (superficial); IM, intestinal metaplasia; EECs, enteroendocrine cells; PMCs, pit mucous cells; GMCs, gastric mucous cells.

Monocle2 reconstructed a bifurcated epithelial backbone along GMC and pit mucous cell (PMC) lineages (Fig 3A and 3B). Chief cells, EEC, and parietal cells mostly occupied early pseudotime, malignant epithelium 1 spanned early-to-late stages, and malignant epithelium 2 concentrated at late endpoints; stem-like epithelium was distributed throughout, indicating multi-origin/multi-path replenishment. State-wise enrichment in malignant epithelium showed (i) cellular components enriched for lumenal structures, junctional complexes, and mitochondrial ribosomes (Fig 3C); (ii) biological processes enriched for mitochondrial biogenesis/metabolism, electron transport/respiration, and RNA splicing (Fig 3D); (iii) molecular functions enriched for ubiquitin-related binding and electron-transport regulation (Fig 3E); and (iv) KEGG pathways highlighting cell adhesion/tight junctions, apoptosis, lymphocyte migration, cellular senescence, along with glycolysis/gluconeogenesis, TP53, HIF-1, ferroptosis, necroptosis, and mitophagy (Fig 3F). These data indicate that late-stage malignant evolution couples metabolic rewiring with adhesion/junction remodeling and stress–death pathway re-balancing, cohering with the enhanced inflammation/ECM signaling in the communication network.

### 3.4 Transcriptional regulatory features of malignant epithelium

**Fig 4:**
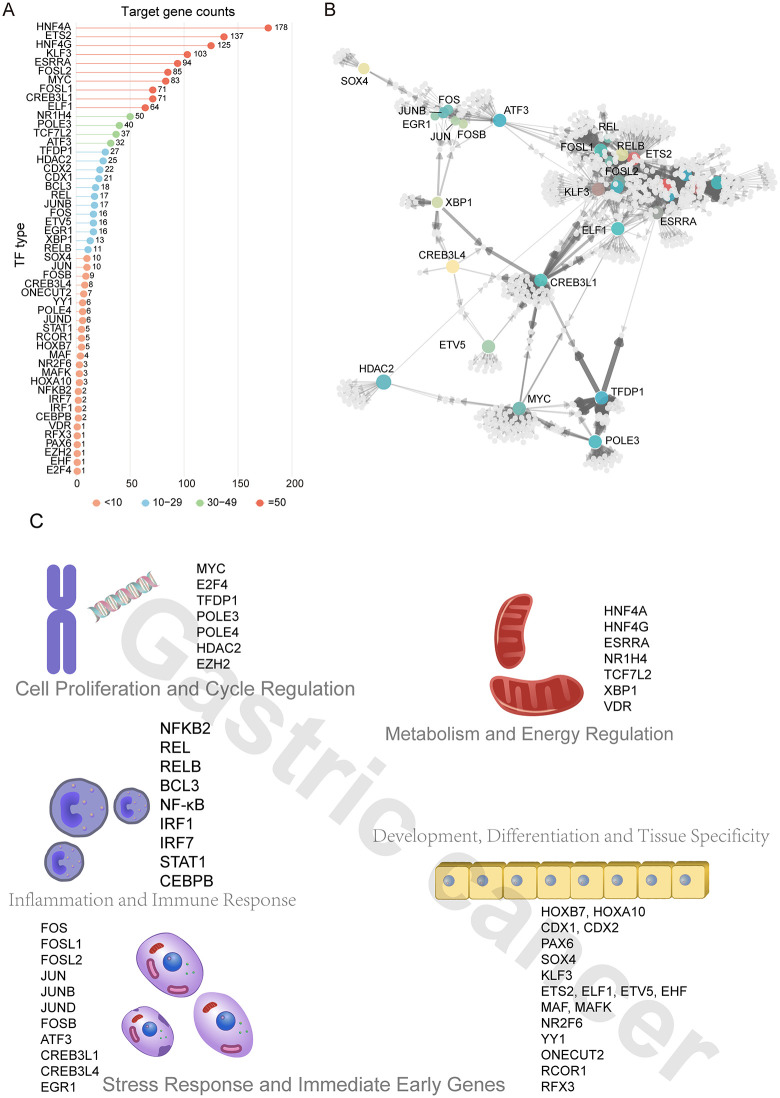
Transcription-factor regulatory landscape of epithelial development in tumors. **(A)** Bar plot of significant TF regulons identified by SCENIC; numbers indicate the size of each regulon (downstream targets). **(B)** Network view of TF regulons controlling >10 targets, highlighting TF–TF connectivity. TFs are colored nodes with labels; downstream target genes are light-gray nodes; arrows indicate regulatory direction; edge width reflects inferred regulatory strength. **(C)**, classify transcription factors into five categories based on their functions.

Based on transcription factor analysis of malignant epithelial cells and other non-malignant epithelial cells (adjacent normal, IGS, IM) from GC tissues, we identified 51 significant transcription factors that participate in the development and progression of epithelial cells. Among them, HNF4A/HNF4G, ETS2, and KLF3 regulated the most abundant target genes, each exceeding 100 targets. Additionally, transcription factors including ESRRA, FOSL2, MYC, FOSL1, CREB3L1, ELF1, and NR1H4 each regulated more than 50 target genes. We ranked all significant transcription factors by the number of regulated target genes in descending order (Fig 4A). To further explore the regulatory relationships between transcription factors and their target genes, we constructed a transcription factor-target gene interaction network (Fig 4B). The results revealed extensive crosstalk among target genes regulated by different transcription factors. Notably, target genes regulated by CREB3L1 played a central role, mediating interactions with multiple other transcription factors. Meanwhile, members of the FOS and JUN families exhibited a clustered distribution pattern in the network, suggesting that target genes regulated by these transcription factors share similar biological functions. Based on their biological functions, we classified these 51 transcription factors into five major categories (Fig 4C): Metabolism and Energy Regulation, Cell Proliferation and Cycle Regulation, Inflammation and Immune Response, Stress Response and Immediate Early Genes, and Development, Differentiation and Tissue Specificity. Among these, transcription factors involved in Development, Differentiation and Tissue Specificity were the most abundant, accounting for over 35%. This finding indicates that the development and progression of malignant gastric epithelial cells are accompanied by significant alterations in cellular differentiation states and remodeling of tissue-specific characteristics, further supporting the notion that epithelial cells undergo complex phenotypic transitions during carcinogenesis.

### 3.5 Cell-specific TFs and their impact on prognosis in GC patients

**Fig 5:**
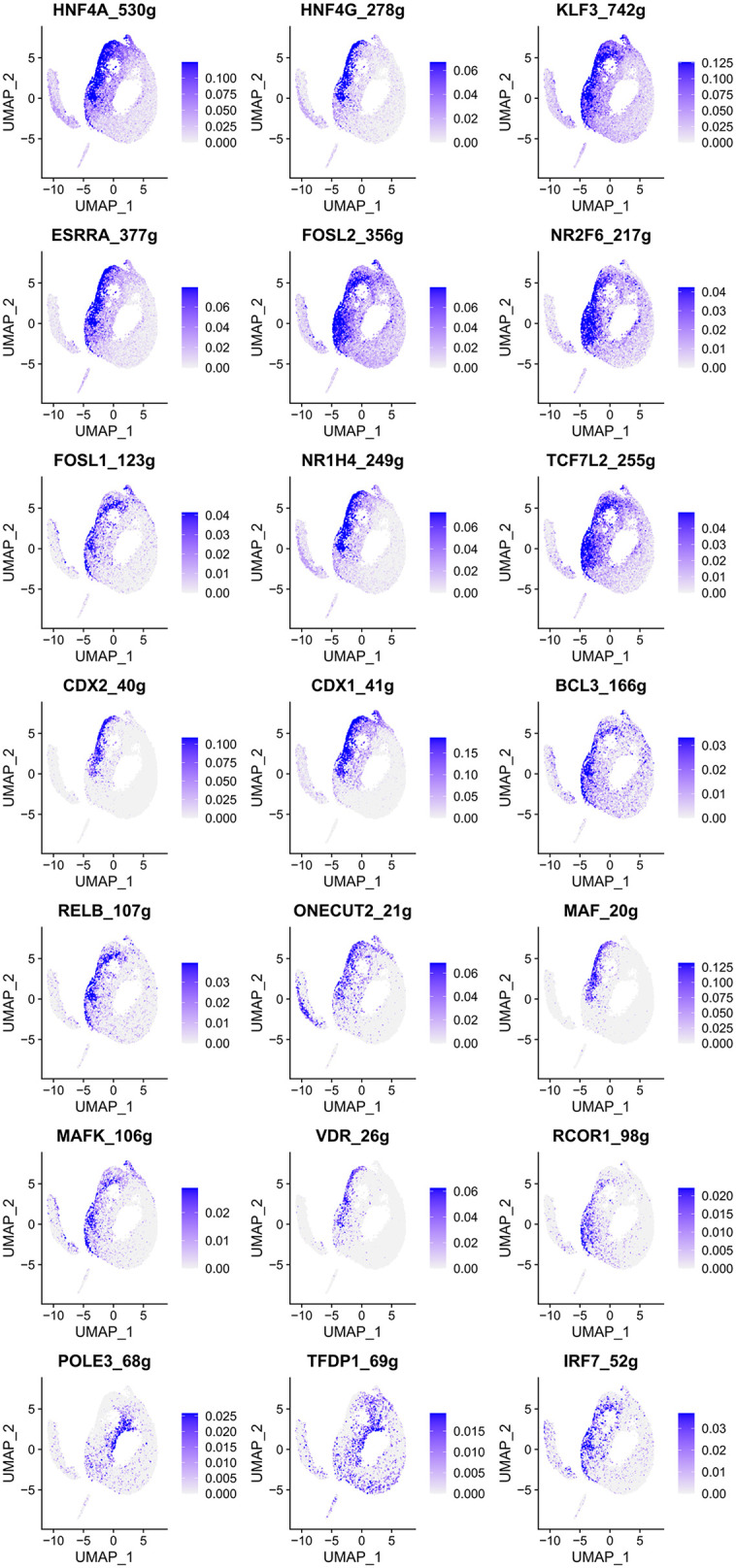
TF regulons with elevated activity in malignant epithelium (SCENIC). UMAPs show the regulon specificity score (RSS) for representative TFs; blue indicates higher RSS within clusters.

**Fig 6:**
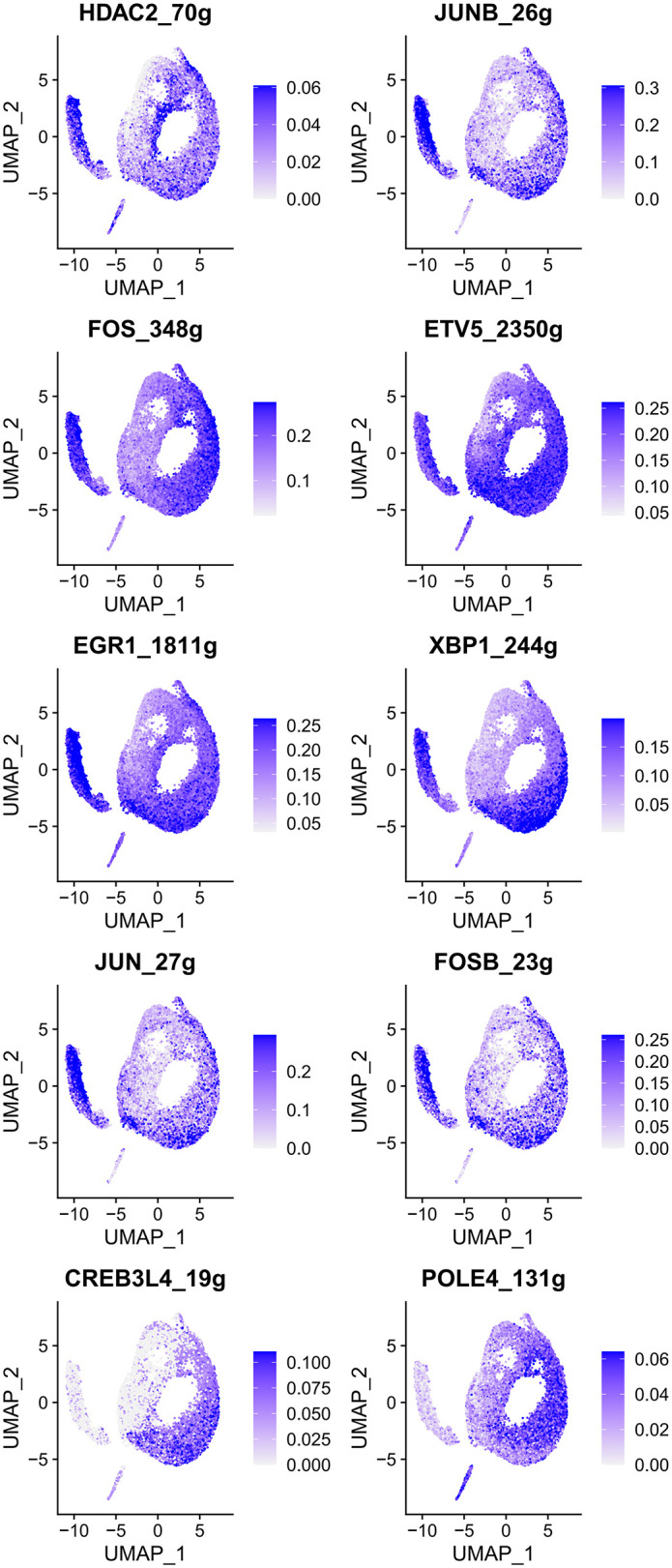
TF regulons suppressed in malignant epithelium (SCENIC). UMAPs of regulon specificity score (RSS) for representative TFs; blue indicates higher RSS values mapped onto clusters (lower activity in malignant epithelium relative to other epithelial states is highlighted by comparative context).

To visualize the activity of TFs across epithelial cell populations, we mapped the regulon activity scores (AUC values) onto the UMAP projection of epithelial cells. Our analysis revealed 21 TFs exhibiting distinctively high activity scores in malignant gastric epithelial cells (Fig 5), including HNF4A_530g, HNF4G_278g, KLF3_742g, ESRRA_377g, FOSL2_356g, NR2F6_217g, FOSL1_123g, NR1H4_249g, TCF7L2_255g, CDX2_40g, CDX1_41g, BCL3_166g, RELB_107g, ONECUT2_21g, MAF_20g, MAFK_106g, VDR_26g, RCOR1_98g, POLE3_68g, TFDP1_69g, and IRF7_52g. In contrast, 10 TFs showed relatively low activity scores in malignant epithelial cells, namely HDAC2_70g, JUNB_26g, FOS_348g, ETV5_2350g, EGR1_1811g, XBP1_244g, JUN_27g, FOSB_23g, CREB3L4_19g, and POLE4_131g (Fig 6). Additionally, 18 TFs, including ETS2, CREB3L1, ELF1, ATF3, MYC, JUND, EHF, YY1, SOX4, RFX3, STAT1, REL, NFKB2, IRF1, CEBPB, PAX6, EZH2, and E2F4, exhibited relatively balanced activity scores between non-malignant and malignant epithelial cells, displaying group-dependent modulation across epithelial subsets (Figure S2 in S3 File).

**Fig 7:**
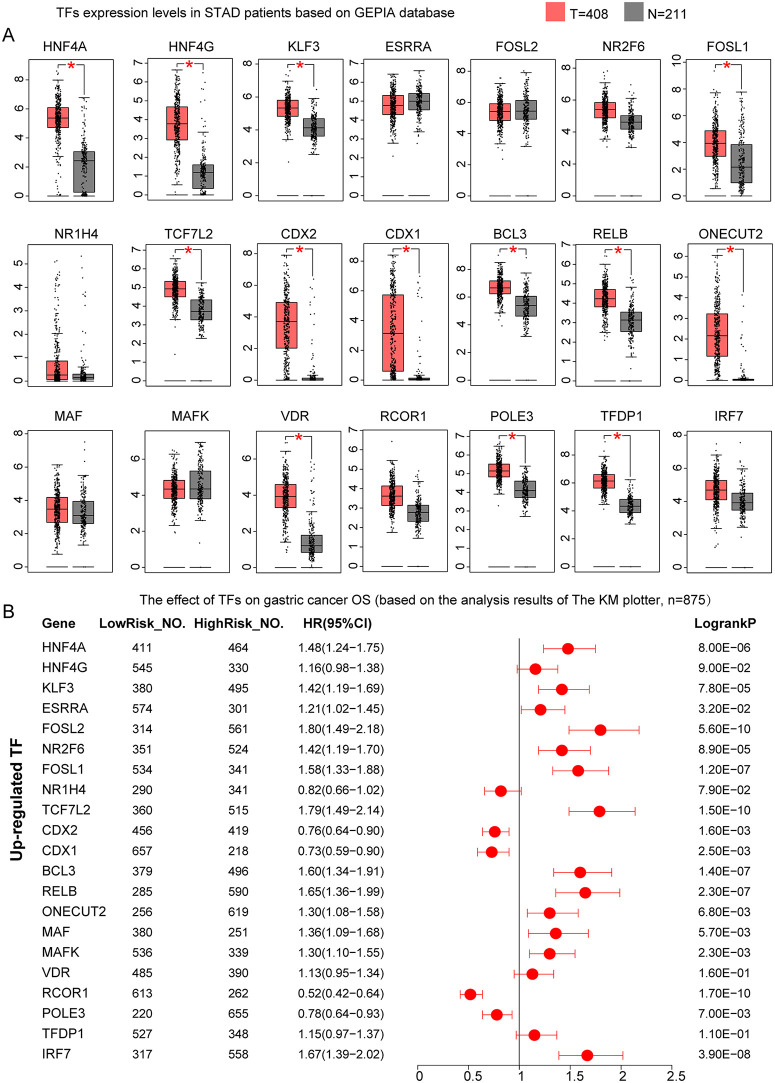
Expression profiles and prognostic significance of transcription factors with elevated activity in malignant epithelial cells of gastric cancer. **(A)** Differential expression analysis of transcription factors between tumor and normal tissues using the GEPIA database. **(B)** Forest plot of Kaplan-Meier plotter-based survival analysis demonstrating the prognostic impact of transcription factor expression levels in gastric cancer patients. Red dots represent hazard ratio (HR) values; horizontal red lines indicate 95% confidence intervals (CI), with lower and upper limits on the left and right, respectively. LowRisk_NO., number of samples with low TF expression; HighRisk_NO., number of samples with high TF expression. *, P < 0.05. T, tumor sample size; N, normal sample size.

To further evaluate the clinical relevance of these TFs in GC, we validated their expression levels and prognostic impact using external datasets. Analysis of TCGA-based GC patient data from the GEPIA database revealed that HNF4A, HNF4G, KLF3, FOSL1, TCF7L2, CDX2, CDX1, BCL3, RELB, ONECUT2, VDR, POLE3, and TFDP1 were significantly upregulated in GC tissues (P < 0.05, Fig 7A). Survival analysis based on GC microarray data demonstrated that high expression of HNF4A, KLF3, FOSL1, TCF7L2, BCL3, RELB, and ONECUT2 was significantly associated with poor prognosis (HR > 1, P < 0.05), whereas elevated expression of CDX2, CDX1, and POLE3 correlated with favorable outcomes in GC patients (HR < 1, P < 0.05, Fig 7B). Moreover, although ESRRA, FOSL2, NR2F6, MAF, MAFK, VDR, and IRF7 showed no statistically significant differential expression between tumor and normal tissues (P > 0.05), their elevated expression levels were associated with unfavorable prognosis in GC (HR > 1, P < 0.05, Fig 7B). Multi-gene survival analysis using the GEPIA3 platform further confirmed that high expression of MAF and ONECUT2 was significantly associated with poor prognosis in GC (HR > 1, P < 0.05, Figure S3A in S3 File).

**Fig 8:**
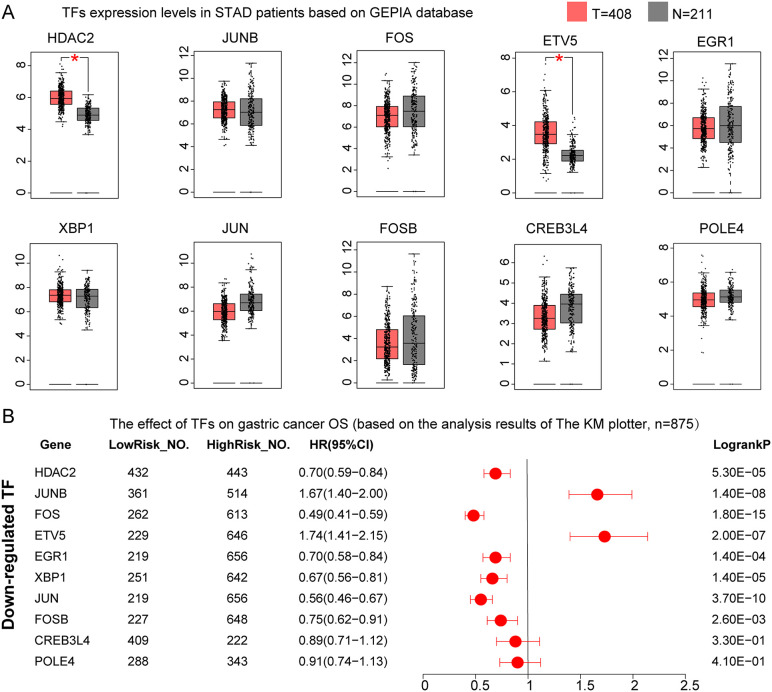
Expression profiles and prognostic significance of transcription factors with reduced activity in malignant epithelial cells of gastric cancer. **(A)** Differential expression analysis of transcription factors between tumor and normal tissues using the GEPIA database. **(B)** Forest plot of Kaplan-Meier plotter-based survival analysis demonstrating the prognostic impact of transcription factor expression levels in gastric cancer patients. Red dots represent hazard ratio (HR) values; horizontal red lines indicate 95% confidence intervals (CI), with lower and upper limits on the left and right, respectively. LowRisk_NO., number of samples with low TF expression; HighRisk_NO., number of samples with high TF expression. *, P < 0.05. T, tumor sample size; N, normal sample size.

Among the 10 TFs with reduced activity scores in malignant epithelial cells compared to non-malignant cells (HDAC2, JUNB, FOS, ETV5, EGR1, XBP1, JUN, FOSB, CREB3L4, and POLE4), only HDAC2 and ETV5 exhibited significantly elevated expression in GC tissues (P < 0.05, Fig 8A). Interestingly, high expression of HDAC2, FOS, EGR1, XBP1, JUN, and FOSB conferred a protective effect on patient survival (HR < 1, P < 0.05, Fig 8B), while elevated expression of ETV5 and JUNB was associated with increased risk (HR > 1, P < 0.05, Fig 8B). Multi-gene survival analysis in GEPIA3 showed no significant prognostic association for these 10 TFs collectively (P > 0.05, Figure S3B in S3 File).

**Fig 9:**
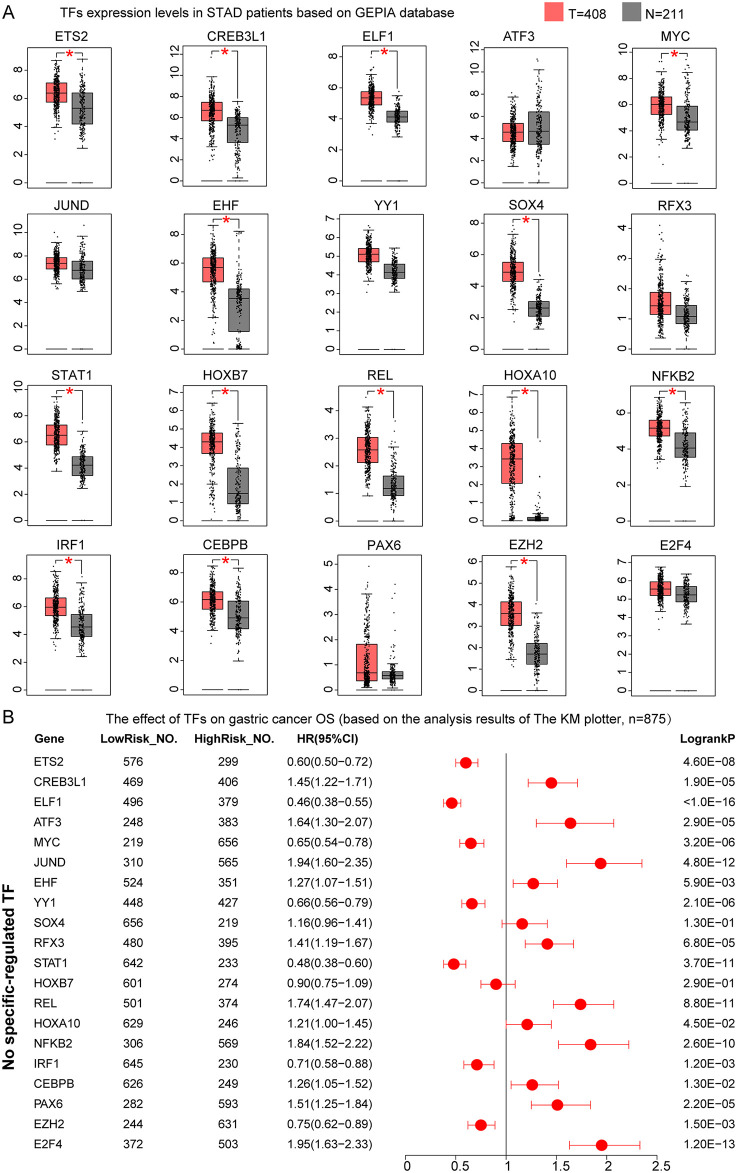
Expression profiles and prognostic significance of transcription factors with comparable activity between malignant and non-malignant epithelial cells of gastric cancer. **(A)** Differential expression analysis of transcription factors between tumor and normal tissues using the GEPIA database. **(B)** Forest plot of Kaplan-Meier plotter-based survival analysis demonstrating the prognostic impact of transcription factor expression levels in gastric cancer patients. Red dots represent hazard ratio (HR) values; horizontal red lines indicate 95% confidence intervals (CI), with lower and upper limits on the left and right, respectively. LowRisk_NO., number of samples with low TF expression; HighRisk_NO., number of samples with high TF expression. *, P < 0.05. T, tumor sample size; N, normal sample size.

Among the 20 TFs displaying non-distinctive activity patterns between malignant and non-malignant epithelial cells, ETS2, CREB3L1, ELF1, MYC, EHF, SOX4, STAT1, HOXB7, REL, HOXA10, NFKB2, IRF1, CEBPB, and EZH2 were highly expressed in GC tissues (Fig 9A). Survival analysis revealed that high expression of CREB3L1, EHF, REL, HOXA10, NFKB2, and CEBPB, as well as ATF3, JUND, RFX3, PAX6, and E2F4 (which showed no significant upregulation in tumor tissues), were significantly associated with poor prognosis. Conversely, elevated expression of ETS2, ELF1, MYC, YY1, STAT1, IRF1, and EZH2 conferred protective effects on patient survival (Fig 9B). Multi-gene survival analysis in GEPIA3 confirmed that E2F4, ATF3, STAT1, and CREB3L1 were associated with unfavorable prognosis, while EZH2 and HOXA10 correlated with favorable outcomes in the TCGA database (Figure S3C in S3 File).

### 3.6 Validation of candidate TFs across primary and metastatic lesions

**Fig 10:**
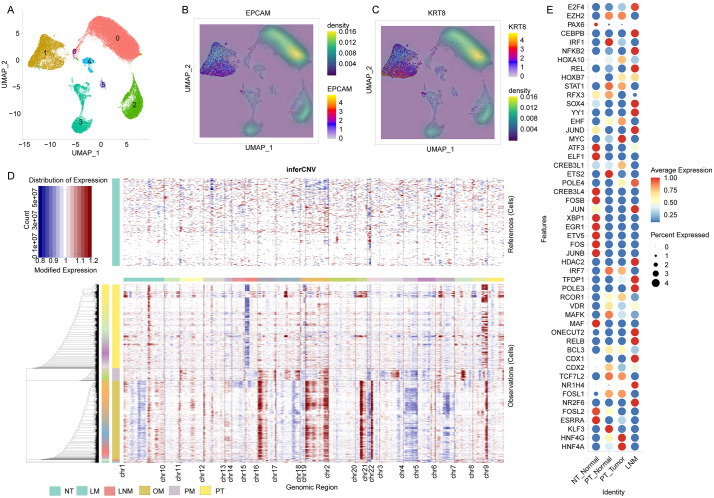
Independent single-cell validation of epithelial malignancy and inferred copy-number alterations in the GSE163558 cohort. **(A)** Uniform Manifold Approximation and Projection (UMAP) visualization of 53,338 cells from GSE163558 clustered using PC = 10 and resolution = 0.02, yielding seven major cell clusters. **(B-C)** FeaturePlot showing expression of EPCAM **(B)** and KRT8 **(C)**, highlighting the epithelial-enriched cluster. **(D)** inferCNV heatmap of epithelial cells using epithelial cells from the normal tissue as the reference, illustrating inferred copy-number alteration (CNV) profiles across epithelial cells derived from primary tumors (PT) and metastatic lesions. **(E)** Dotplot for the candidate TFs in different groups from GSE163558 cohort. NT, normal tissues; LM, liver metastasis; LNM, lymph node metastasis; OM, ovarian metastasis; PM, peritoneal metastasis; PT, primary tumor.

In the GC scRNA-seq dataset GSE163558, a total of 53,338 cells were analyzed and clustered into seven major cell populations using principal components 10 and a clustering resolution of 0.02 (Fig 10A). Cluster 1 (n = 9,675) exhibited markedly elevated expression of EPCAM (Fig 10B) and KRT8 (Fig 10C), supporting its annotation as epithelial cells. These epithelial cells were extracted for CNV inference using inferCNV, with epithelial cells from the normal tissue sample serving as the reference. The inferred CNV profiles demonstrated prominent CNV alterations in epithelial cells derived from both primary tumors and metastatic lesions (Fig 10D), with the ovarian metastasis displaying the most pronounced CNV signatures. We next stratified epithelial cells from primary tumors into normal-like epithelial cells and putative malignant epithelial cells according to inferCNV results, and further incorporated epithelial cells from the LNM group to validate the expression patterns of candidate transcription factors. DotPlot visualization (Fig 10E) revealed that FOSB, JUN, XBP1, EGR1, ETV5, FOS, and JUNB were highly expressed in normal gastric epithelial cells, whereas other candidate transcription factors showed varying degrees of upregulation in non-normal epithelial subsets. Collectively, these observations were consistent with our primary findings and further support the potential involvement of these transcription factors in GC initiation and progression.

## 4. Discussion

In a unified analytical framework spanning CGS, IM, cancer-adjacent, and tumor tissues, we assembled a cross-stage scRNA-seq atlas of GC and delineated the emergence and progression-associated changes of malignant epithelium with multi-layered evidence. Within epithelial cells, clustering plus inferCNV segregated normal, evolving (proto-malignant), and malignant states, suggesting broad chromosomal instability in tumors and revealing two biologically distinct malignant subgroups—cluster 1, retaining epithelial-differentiation features, and cluster 2, enriched for inflammation/EMT and antigen-presentation programs. In parallel, tumors exhibited a community-level reorganization characterized by epithelial contraction alongside immune/stromal expansion; CellChat demonstrated globally intensified predicted epithelial communications with T cells, myeloid cells, and fibroblasts, with the CD44 adhesion–ECM axis emerging as a prominent signaling pathway. Monocle2 trajectories mapped epithelial differentiation along GMC and PMC backbones, positioning malignant subsets at later pseudotime correlating with elevated CNV burden, while stem-like epithelium persisted across stages with potential progenitor characteristics. SCENIC further revealed multi-axis regulatory alterations involving HNF4/CDX, NR1H4/ESRRA, MYC/TFDP1, and FOSL1/REL/NFKB, accompanied by reduced activity of JUN/XBP1-linked response programs. Together, these findings support a three-tier progression-associated pattern—epithelial trajectory reconfiguration → immune/stromal remodeling → transcriptional-network reprogramming—that provides observational evidence consistent with malignant gastric epithelium progression toward invasive phenotypes and highlights potential translational opportunities, including the CD44 axis and specific TF regulon modules as candidate biomarkers and therapeutic entry points.

Our multi-layer analyses reveal patterns consistent with a model in which malignant gastric epithelium progresses along an intestinalization–inflammation/EMT continuum, potentially supported by immune–stromal crosstalk centered on adhesion/ECM signaling. First, the malignant clusters that we define by high CNV and late pseudotime preferentially activate HNF4/CDX intestinal programs together with nuclear-receptor and metabolic regulators (e.g., NR1H4/FXR, ESRRA), consistent with prior evidence that HNF4A can activate CDX2 and functionally bridge IM and GC, and that bile-acid receptors (TGR5–HNF4A/FXR) promote IM-like reprogramming under chronic exposure [31,32]. These data suggest that intestinal differentiation modules may actively contribute to malignant identity in a subset of tumors. Second, our CellChat results predict CD44-centered adhesion as a prominent hub of epithelial communication with T cells, myeloid cells, and fibroblasts—aligning with reports that CD44 marks aggressive/CSC-like fronts and associates with adverse prognosis in GC [33–35]. Third, malignant subgroup C2 shows reinforced inflammation and EMT signature, matching canonical roles of EMT in invasion and immune evasion; this co-occurs with a TME that single-cell atlases have shown to be rich in CAF and TAM programs with pro-tumorigenic signals. Together, these strands are consistent with a model in which intestinalized malignant epithelium (HNF4/CDX/FXR axis) may be amplified by CAF/TAM-rich niches via adhesion/ECM and chemokine circuits (CD44 and allied pathways), potentially facilitating transitions toward EMT-like, invasive states.

Disentangling benign inflammation from proto-malignancy is notoriously difficult in GC. Here, inferCNV provided an orthogonal, genome-scale signal to stratify epithelial cells into normal, evolving, and malignant states, which we then cross-validated by pseudotime (Monocle2), intercellular signaling (CellChat), and regulon activity (SCENIC). This consilience of evidence is critical: inferCNV separates copy-number–driven states, Monocle2 positions them along branching trajectories that resolve early–late transitions, CellChat quantifies how malignant subsets rewire predicted outgoing and incoming signals, and SCENIC exposes TF modules (HNF4/CDX, NR1H4/ESRRA, MYC/TFDP1, FOSL1/REL/NFKB, FOS/JUN) that are associated with the transcriptional reprogramming we observe. Notably, the CD44 axis emerges across modalities—as a communication hub (CellChat), as a marker of invasive fronts in prior GC studies, and as a pathway coherently elevated with EMT/inflammation in the C2 malignant subgroup—strengthening its candidacy as a translational target [36–39]. Importantly, our choice of widely adopted, peer-reviewed methods (inferCNV, CellChat, Monocle2, SCENIC) rests on extensive validation in single-cell oncology and enhances the robustness and interpretability of regulon-level inferences. Taken together, the method-mechanism synergy here—CNV states → trajectory stage → signaling rewiring → TF regulons—offers a coherent correlative framework for GC progression and a practical blueprint for biomarker/target nomination.

Our SCENIC results delineate a multi-axis regulatory alteration in malignant epithelium, consolidating the five functional TF categories into four interconnected regulatory modules that capture the major biological processes driving malignant progression: the intestinal metabolic and differentiation axis (HNF4A/HNF4G–CDX1/2) [40,41], the metabolic/nuclear-receptor axis (NR1H4/FXR–ESRRA) [42–44], the proliferation axis (MYC/TFDP1–E2F) [45], and the inflammation/EMT axis (FOSL1/AP-1–REL/NF-κB) [46,47]. Prior functional studies demonstrate that HNF4A activates CDX2 via a shadow enhancer and recruits intestinal enhancers in gastric cells, providing a TF hierarchy that bridges IM to cancer [32]; concordantly, HNF4A is elevated in IM and GC and associates with poorer outcomes, consistent with its potential role as an upstream driver rather than a passive marker. On the metabolic front, FXR/NR1H4 [48,49] and bile-acid signaling—including the TGR5–HNF4α axis [50]—induce IM-like programs and lipid metabolic rewiring, mirroring our SCENIC activation of NR1H4/ESRRA and implicating chronic bile-acid exposure in lineage/metabolic drift toward malignant states. Concurrently, heightened MYC/TFDP1–E2F activity aligns with cell-cycle acceleration and replication stress; multi-cohort GC analyses underscore the prognostic and oncogenic roles of E2Fs [51–53], reinforcing the proliferative axis identified by SCENIC. At the invasion–immunity interface, the upregulation of FOSL1(AP-1) and REL/NF-κB modules coheres with canonical EMT, inflammatory signaling, and immune evasion; prior work shows FOSL1 drives migration/EMT, while NF-κB serves as a central hub of inflammation and tumor progression—precisely the pattern we observe in the inflammation/EMT-enriched malignant subgroup [46,54–56]. Collectively, SCENIC reveals a progression-associated regulatory pattern—intestinalization (HNF4/CDX) → metabolic nuclear-receptor signaling (FXR/ESRRA) → proliferative gain (MYC/TFDP1–E2F) → inflammatory/EMT programs (FOSL1/REL–NF-κB)—that dovetails with recent single-cell GC atlases linking TME remodeling to transcriptional state transitions, and identifies HNF4A–CDX2, FXR/NR1H4, and FOSL1/NF-κB modules as candidate biomarkers and therapeutic entry points.

The clinical significance of this study lies in integrating multiple lines of single-cell evidence—inferCNV, CellChat, Monocle2, and SCENIC—within a unified framework to characterize a continuum from proto-malignant to malignant epithelium, to identify regulatory axes (e.g., HNF4/CDX, NR1H4/ESRRA), and to highlight CD44–ECM–centered immune/stromal crosstalk; together these features yield actionable molecular readouts for early warning and risk-stratified surveillance in high-risk CGS/IM populations (e.g., combining CNV scores with core regulon activities) and offer a mechanistic rationale for treatment strategies aligned with malignant epithelial classes C1 (differentiation-biased) and C2 (inflammation/EMT-enhanced), including rational combinations of anti-adhesion/anti-stroma approaches, nuclear-receptor modulation, anti-inflammatory/anti-EMT interventions, and immunotherapy. Importantly, external validation using GEPIA and KM plotter databases revealed that malignant-epithelium-enriched transcription factors exhibited diverse prognostic associations in GC. Among TFs with elevated activity in malignant cells, high tissue-level expression of HNF4A, KLF3, FOSL1, TCF7L2, BCL3, RELB, ONECUT2, and MAF correlated with poor OS, whereas CDX2, CDX1, and POLE3 were associated with favorable outcomes, indicating context-dependent and potentially stage-specific roles of these regulatory programs. Notably, several TFs—including ESRRA, NR2F6, MAFK, and IRF7—showed no significant differential expression between tumor and normal tissues yet their elevated levels predicted adverse prognosis, while others such as HDAC2, XBP1, and JUN exhibited protective effects despite being downregulated TF activity in malignant epithelium. These patterns suggest a dual nature of transcriptional regulation in gastric carcinogenesis: on one hand, malignant cells acquire tumor-specific regulatory circuits (e.g., HNF4A/CDX, FOSL1/NF-κB axes) that drive progression; on the other hand, they retain or aberrantly activate certain transcriptional features of normal epithelium, whose prognostic impact may reflect residual differentiation capacity or compensatory stress responses.

However, we acknowledge several important limitations. First, the lack of systematic incorporation of clinical covariates—including tumor stage, grade, patient sex, and age—represents a significant limitation. These clinical features are known to influence tumor heterogeneity, cell composition, signaling patterns, and genomic alterations in GC. The absence of stratification or adjustment for these variables may confound our findings and limit their generalizability across different patient subgroups. Future studies should explicitly integrate and control for these clinical parameters to determine whether the identified cellular states and regulatory networks exhibit differential patterns across clinical strata. Second, the cross-sectional design and sampling heterogeneity inherently limit causal inference. Third, despite rigorous quality control to remove RNA contamination and doublets, and our selection of typical GC tumor cells for inferCNV analysis, algorithmic biases inherent to computational CNV inference from transcriptomic data remain unavoidable. These include potential bias from using CGS T cells as the inferCNV reference, sensitivity to gene expression variability, and the probabilistic nature of CNV calls; reliance on transcriptome-derived CNV inferences without matched WES/WGS or FISH validation further limits the precision of copy number estimates. Fourth, the probabilistic, motif-dependent nature of CellChat and SCENIC introduces possible false positives and undirected edges. Fifth, trajectory reconstruction and cross-cohort integration are sensitive to parameter choices and batch effects. Finally, the absence of causal functional validation (organoids/CRISPR/spatial multi-omics) and external cohorts linking findings to treatment response constrains the translational interpretation of our results. Accordingly, these results should be regarded as hypothesis-generating observations that warrant longitudinal, spatially resolved, and perturbation-based validation.

## 5. Conclusion

By integrating scRNA-seq profiles spanning CGS, IM, cancer-adjacent, and tumor tissues, this study delineates a progression-associated continuum characterized by epithelial trajectory reconfiguration, immune and stromal compartment remodeling, and transcriptional-network reorganization. Malignant epithelial cells preferentially occupy late-pseudotime positions along GMC/PMC differentiation backbones, coinciding with elevated inferred CNV burden and augmented predicted intercellular communication with T cells, myeloid cells, and fibroblasts—particularly through a CD44–ECM axis. Gene regulatory network analysis further reveals coordinated shifts in lineage-specifying (HNF4A, HNF4G, CDX2), metabolic (NR1H4, ESRRA), proliferative (MYC, TFDP1), and inflammatory/EMT-associated (FOSL1, REL, NF-κB family) regulon activities. These findings are consistent with a model in which an inflammatory and intestinalized mucosal background may facilitate progression toward invasive GC phenotypes, and they nominate a set of candidate transcriptional regulators for prospective evaluation as early-detection biomarkers and potential therapeutic targets.

## Supporting information

S1 File Supplementary methods. (DOCX)

S2 File Supplementary code. (ZIP)

S3 File Supplementary figures. (DOCX)
