# Supplementary material for: Single-cell atlas of gastric cancer reveals malignant epithelial evolution and regulatory reprogramming of the tumor microenvironment
Source: PLoS One. 2026 Apr 22;21(4):e0347679. doi: 10.1371/journal.pone.0347679 (PMC13102225; doi:10.1371/journal.pone.0347679)
Supplement: S1 File — (DOCX) [file pone.0347679.s001.docx]

**Single-Cell Atlas of Gastric Cancer Reveals Malignant Epithelial Evolution and Regulatory Reprogramming of the Tumor Microenvironment**

Xiulan Li ^1^, Mengqi Guo ^2^, Yunhan Wen ^2,*^, Bo Long ^3,*^

^1^ Department of Gastroenterology, First Affiliated Hospital of Hunan Normal University & Hunan Provincial People's Hospital, Changsha 410001, Hunan, China

^2^ Research Institute of Lanzhou University in Shenzhen, Lanzhou University, Shenzhen 518063, Guangdong, China.

^3^ Department Three of General Surgery, The Second Hospital & Clinical Medical School, Lanzhou University, Lanzhou, 730000, Gansu, China.

Correspondences:

Yunhan Wen, Email: yunhanwen2014@163.com;

Bo Long, Email: longbo_107@163.com.

## Methods

## Copy Number Variation (CNV) Inference Analysis

### Data Preprocessing and Quality Control

Epithelial cells were first extracted from the complete single-cell dataset for subsequent analysis. To ensure data quality, we implemented a two-step quality control strategy:

**(1) Ambient RNA Contamination Removal**

We employed the decontX algorithm (version 0.99.3) to detect and remove ambient RNA contamination in epithelial cells. DecontX utilizes a Bayesian-based method to estimate the contamination score for each cell, which represents the proportion of transcripts derived from ambient RNA in the cellular transcriptome. Contamination scores were calculated for all epithelial cells and visualized through UMAP projection to display the spatial distribution of contamination levels. To retain high-quality cells, we set a contamination threshold of 0.2, filtering out cells with contamination scores ≥ 0.2. This process resulted in the removal of approximately 20,000 highly contaminated cells.

**(2) Doublet Identification and Removal**

Technical doublets were identified using the DoubletFinder algorithm (version 2.0.3). First, parameter sweeping was performed using the paramSweep_v3 function with a grid search of pN and pK parameters (PCs = 1:20), generating artificial doublets and calculating the pANN (proportion of artificial nearest neighbors) value for each cell. Subsequently, the summarizeSweep function was used to aggregate the sweep results, and the find.pK function determined the optimal pK parameter by selecting the value corresponding to the maximum BCmetric.

Prior to doublet detection, we performed UMAP dimensionality reduction and clustering analysis using the first 20 principal components from Harmony batch correction (resolution set to 0.02) to obtain cellular cluster information. Based on the clustering results, we estimated the proportion of homotypic doublets using the modelHomotypic function. Assuming a doublet formation rate of 7.5%, we calculated the expected number of doublets and adjusted for the homotypic doublet proportion to obtain the expected number of heterotypic doublets. Finally, doublets were identified using the doubletFinder_v3 function (parameters: PCs = 1:20, pN = 0.25, sct = TRUE), and cells classified as singlets were retained for downstream analysis.

### Reference Cell Selection and Preparation

To accurately infer copy number variations in tumor epithelial cells, chromosomally normal cells were required as references. We extracted T cells from cancer-adjacent gastric mucosa samples (CGS, Cancer-adjacent Gastric mucosa Samples) as the reference cell population. Specifically, we selected T cells that simultaneously met the following criteria: PTPRC expression > 1 and EPCAM expression = 0, ensuring that the selected cells were pure immune cells rather than epithelial cells. These high-quality T cells served as normal reference controls for inferCNV analysis.

### InferCNV Analysis

We utilized the inferCNV software package (version 1.24.0) to infer chromosomal copy number variation patterns in tumor epithelial cells. First, we merged tumor tissue-derived epithelial cells (observation group) with cancer-adjacent tissue-derived high-quality T cells (reference group) to construct the inferCNV analysis object.

Based on the raw counts matrix from the Seurat object, we created a cell annotation file that explicitly labeled the sample type (Cancer or CGS) for each cell. When creating the inferCNV object using the CreateInfercnvObject function, we provided:

- Raw counts matrix
- Cell annotation file
- Gene position ordering file (geneFile.txt, containing chromosomal location information for each gene)
- Reference cell group name (CGS)

InferCNV analysis was executed using the run function with the following key parameter settings:

- cutoff = 0.1: Expression threshold for filtering lowly expressed genes
- cluster_by_groups = TRUE: Clustering performed according to cell groupings
- denoise = TRUE: Denoising applied to CNV signals
- HMM = TRUE: Hidden Markov Model used to predict CNV region boundaries and states
- num_threads = 64: 64 threads utilized for parallel computing to accelerate analysis

Analysis results were output in PDF format, including CNV heatmaps, expression matrices, and phylogenetic trees. By comparing gene expression patterns between tumor epithelial cells and normal reference T cells, the inferCNV algorithm identified chromosomal segment amplifications and deletions, thereby revealing genomic instability characteristics of tumor cells at single-cell resolution.

### InferCNV Results Visualization and Cell Classification

**(1) CNV Results Extraction and Data Preparation**

We extracted the corrected gene expression matrix (expr.data) from the final inferCNV analysis output object (run.final.infercnv_obj). This matrix contained modified expression values across chromosomal regions for all cells, where a value of 1 represents normal copy number, values greater than 1 indicate amplification, and values less than 1 indicate deletion. We separately extracted index positions for reference cells (CGS group) and observation cells (Cancer group), and created a cell annotation dataframe labeling cells as either "normal" or "tumoral".

To ensure genes were properly arranged by chromosomal position in the heatmap, we matched genes in the expression matrix with a gene position file (geneFile.txt), which contains chromosomal location information for each gene. Only genes present in both datasets were retained for subsequent analysis.

**(2) K-means-based Cell Clustering**

To identify cell subpopulations with distinct CNV patterns, we performed unsupervised clustering of cellular CNV profiles using the K-means clustering algorithm (stats package version 4.2.3). We first performed parameter sweeping for different cluster numbers (k = 6 to 12) to determine the optimal number of clusters. For each k value, we executed the following steps:

1. Performed K-means clustering on the transposed expression matrix (setting random seed = 123 to ensure reproducibility)
2. Merged clustering results with cell annotation information
3. Sorted cells by cluster number

**(3) CNV Score Calculation**

To quantitatively assess the CNV level of each cell, we calculated a CNV score. The specific calculation method was as follows: first, we subtracted 1 from the expression matrix (setting normal copy number values to 0), then squared each value, and finally calculated the mean value for each cell. This score reflects the degree to which a cell's genome deviates from the normal state, with higher scores indicating higher CNV levels. We used violin plots to display the distribution of CNV scores across different clusters to assist in determining the optimal cluster number.

**(4) ComplexHeatmap Visualization**

We used the ComplexHeatmap package to generate CNV heatmaps that intuitively displayed copy number variation patterns in cells. The main heatmap settings included:

- **Color scheme**: Three-color gradient (blue #377EB8 for deletions, light gray #F0F0F0 for normal, red #E41A1C for amplifications), corresponding to expression values of 0.4, 1.0, and 1.6, respectively
- **Row annotation**: Left side displaying cell clustering groups (using Accent color scheme) and cell types (tumor cells in red, normal cells in blue)
- **Column annotation**: Top displaying positions of 22 autosomes
- **Grouping settings**: Genes grouped by chromosome with 2mm gaps between chromosomes
- Row and column clustering disabled (cluster_rows = FALSE, cluster_columns = FALSE) to maintain cells arranged by K-means clustering results and genes arranged by chromosomal position

Based on CNV heatmaps and CNV score distributions, we ultimately selected k = 10 as the optimal cluster number, which effectively distinguished cell subpopulations with different CNV patterns.

**(5) Cell Subtype Definition and Annotation**

Based on K-means clustering results and CNV pattern characteristics, we further classified the 10 clusters into 4 cell subtypes:

- **TumorC1**: Comprising clusters 1, 2, and 4, representing tumor cell population 1 with similar CNV patterns
- **NormalC1**: Comprising cluster 3, representing normal cell population 1 with low CNV levels
- **TumorC2**: Comprising cluster 5, representing tumor cell population 2 with distinct CNV patterns
- **NormalC2**: Comprising cluster 6, representing normal cell population 2

For cells not included in the inferCNV analysis (such as cells from adjacent tissues, normal gastric mucosa CGS, and intestinal metaplasia IM), we annotated them as "Normal_cells_Adjacent", "Normal_cells_CGS", and "Normal_cells_IM", respectively, based on their tissue of origin.

**(6) Results Integration and Visualization**

We integrated inferCNV prediction results and CNV scores back into the metadata of the original Seurat object. Through cell barcode matching, CNV scores and cell subtype annotations were added to each cell in the object. Subsequently, we performed UMAP dimensionality reduction again using the first 50 principal components from Harmony batch correction (RunUMAP function) and colored cells by inferCNV-predicted cell subtypes to intuitively display the distribution of cells with different CNV states in low-dimensional space. Additionally, we performed re-clustering using the FindNeighbors and FindClusters functions (resolution set to 0.1) on the integrated data and displayed clustering results and CNV prediction results faceted by sample to assess cellular composition and CNV heterogeneity across different samples.

All key results, including K-means clustering annotations, CNV scores, and final cell subtype predictions, were saved for downstream analyses.

### Software Versions

Key software package versions used in this analysis:

- infercnv: 1.24.0
- decontX: 0.99.3
- DoubletFinder: 2.0.3
- stats: 4.2.3
- MCMCpack: 1.7.0
- Seurat: 4.4.0
- ComplexHeatmap: 2.15.4

We have provided the relevant R code to the editor and further for peer review.
