# Supplementary material for: Single-cell atlas of gastric cancer reveals malignant epithelial evolution and regulatory reprogramming of the tumor microenvironment
Source: PLoS One. 2026 Apr 22;21(4):e0347679. doi: 10.1371/journal.pone.0347679 (PMC13102225; doi:10.1371/journal.pone.0347679)
Supplement: S3 File — (DOCX) [file pone.0347679.s003.docx]

Figures


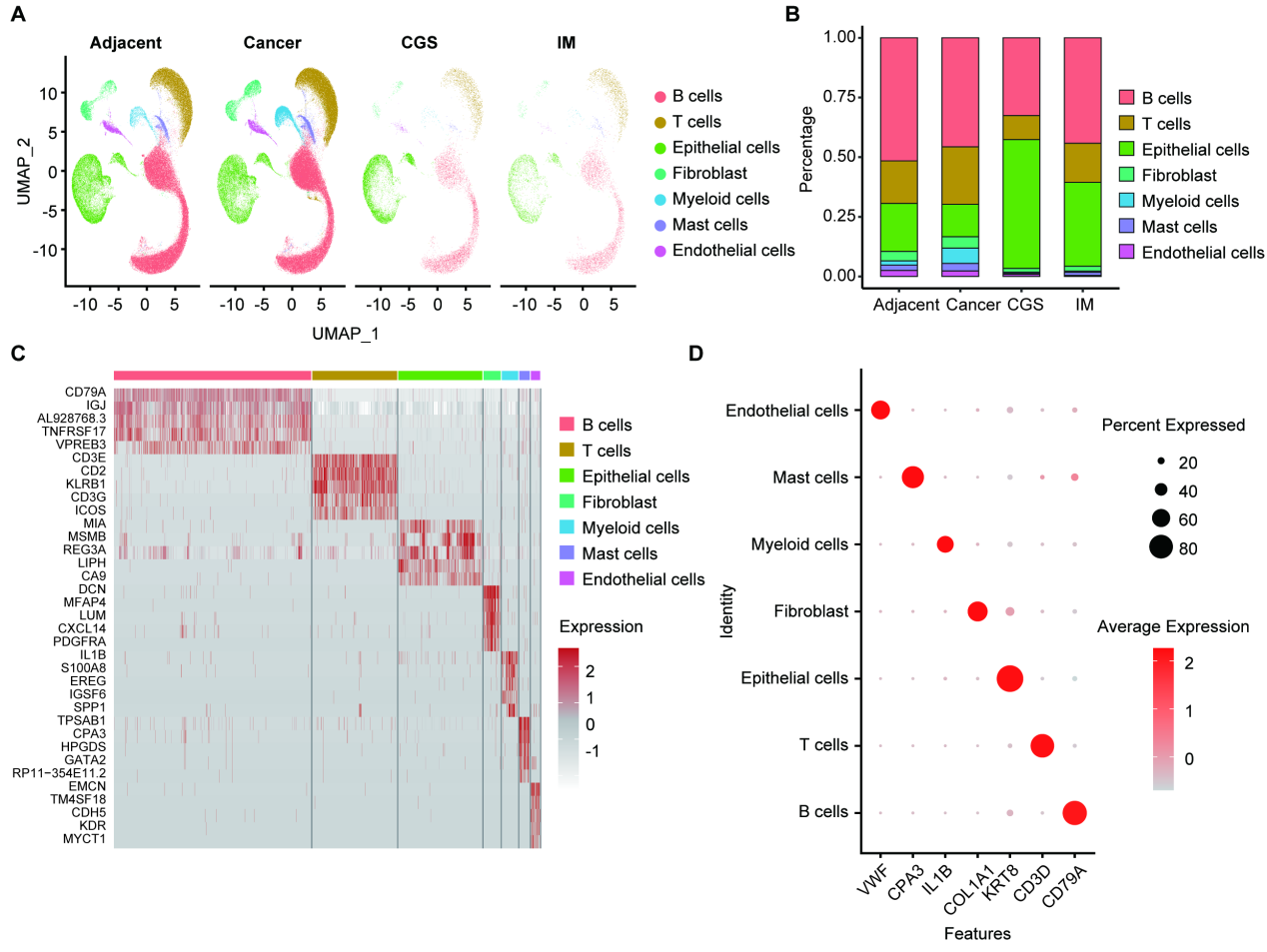
Figure S1. An integrated single-cell atlas across chronic superficial gastritis (CGS), intestinal metaplasia (IM), cancer-adjacent tissue, and gastric tumors.

(A) Distribution of seven major cell types across cancer-adjacent tissue, tumors, CGS, and IM. (B) Cell-type composition (proportions) by tissue category. (C) Heatmap of lineage-specific marker genes for the seven cell types (red indicates higher expression). (D) Bubble plot of representative marker genes for each cell type (red indicates higher expression).


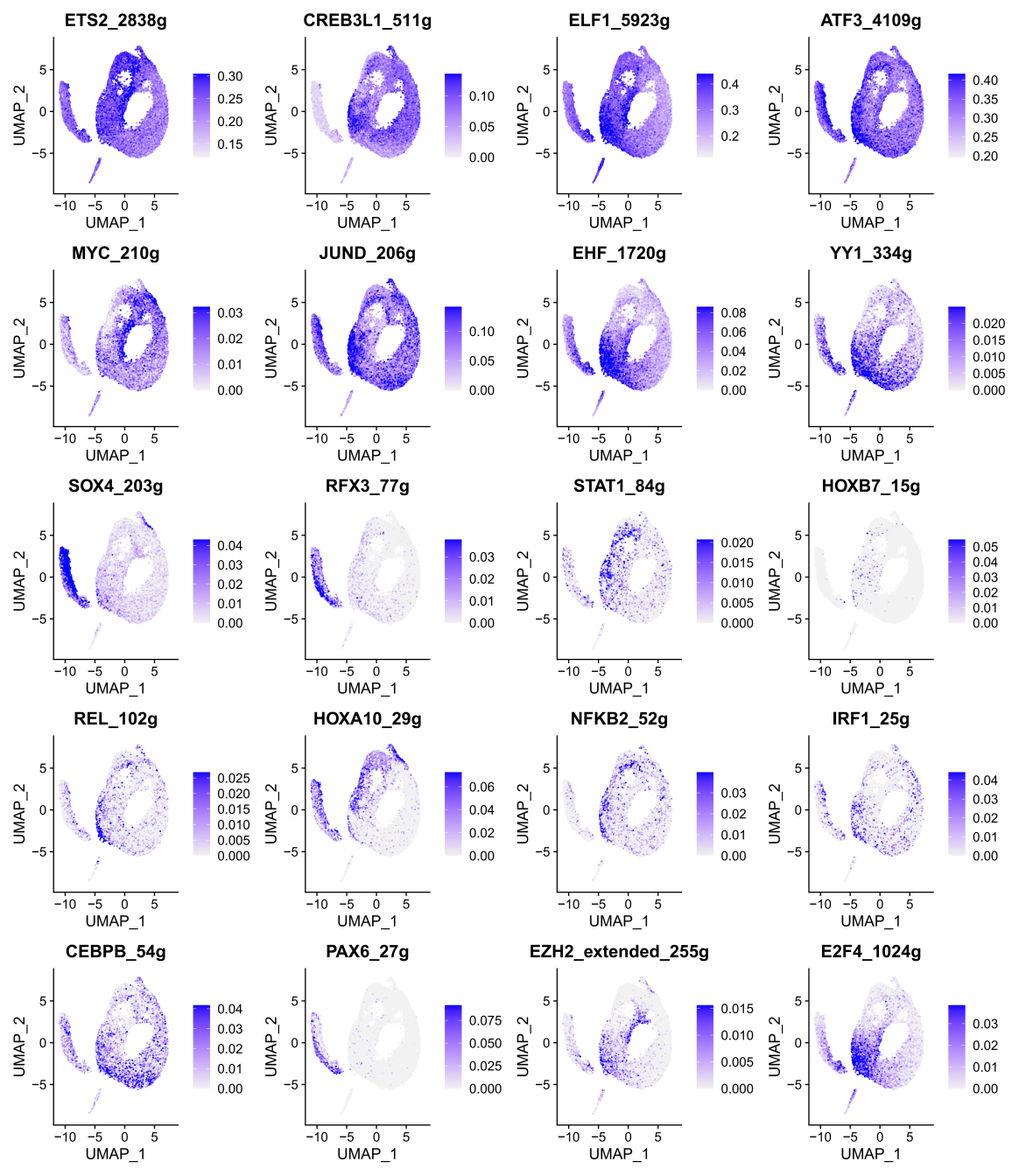
Figure S2. TF regulons ubiquitously expressed across epithelial cells (SCENIC).

UMAPs of RSS for broadly active/condition-dependent TFs in epithelial populations; blue indicates higher RSS within clusters.


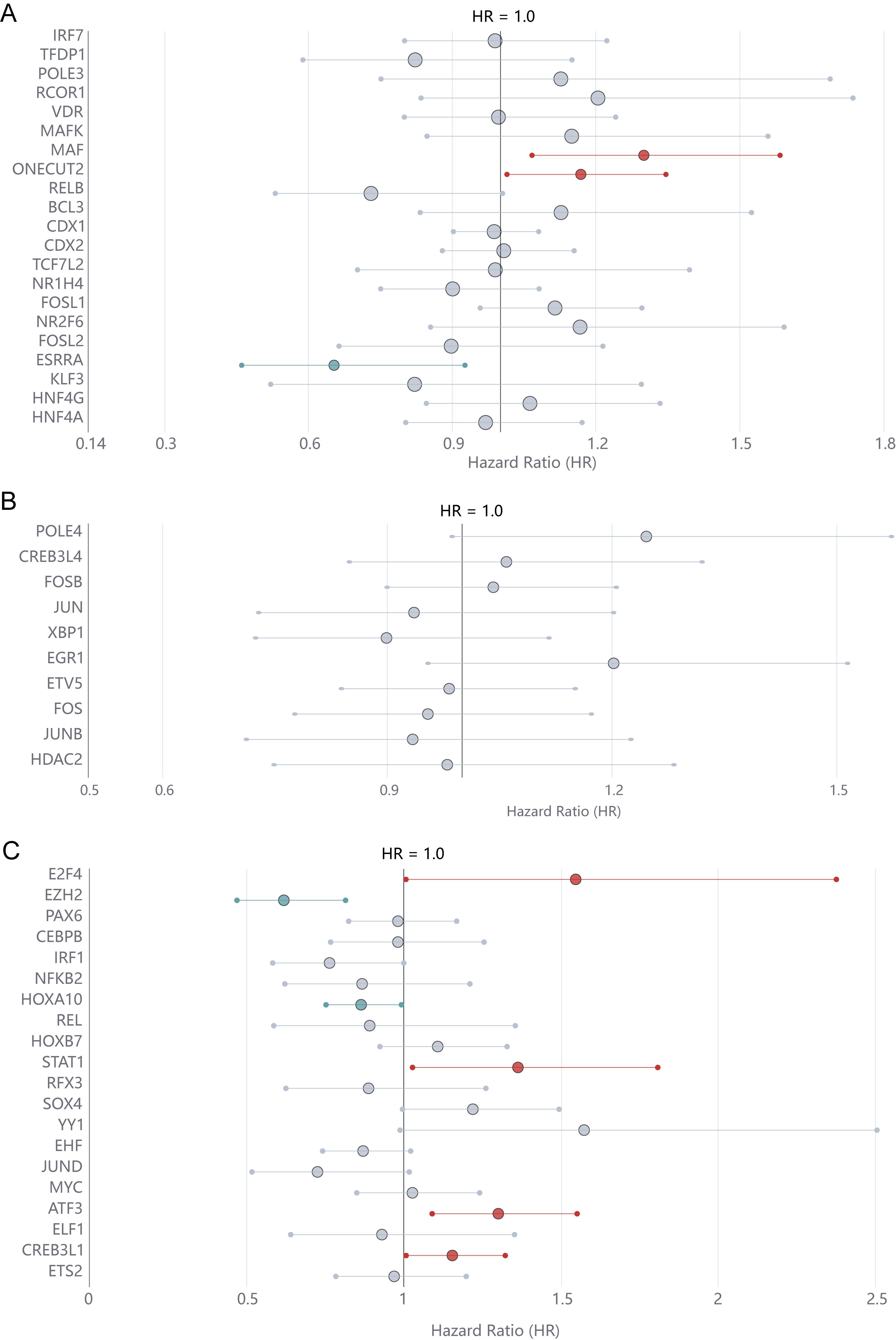


Figure S3 Forest plot of survival analysis for the identified transcription factors based on GEPIA3 database. (A) Transcription factors with elevated activity in malignant epithelial cells. (B) Transcription factors with reduced activity in malignant epithelial cells. (C) Transcription factors showing comparable activity between malignant and non-malignant epithelial cells.
